# Bi-directional modulation of AMPA receptor unitary conductance by synaptic activity

**DOI:** 10.1186/1471-2202-5-44

**Published:** 2004-11-11

**Authors:** Andreas Lüthi, Martin A Wikström, Mary J Palmer, Paul Matthews, Tim A Benke, John TR Isaac, Graham L Collingridge

**Affiliations:** 1 MRC Centre for Synaptic Plasticity, Department of Anatomy, University of Bristol, Bristol, BS8 1TD, UK; 2 Friedrich Miescher Institute, Maulbeerstrasse 66, CH-4058 Basel, Switzerland; 3 Department of Neuroscience, Karolinska institutet, S-171 77 Stockholm, Sweden; 4 MacKay Institute, School of Life Sciences, Keele University, Keele, Staffordshire, ST5 5BG, UK; 5 Pediatrics, Neurology and Pharmacology, UCHSC, 4200 E. 9th Ave, Box B182, Denver, CO 80262 USA; 6 National Institutes of Health, 37 Convent Drive, Building 35/Room 3C-1002, MSC 3701, Bethesda, Maryland 20892–3701, USA

## Abstract

**Background:**

Knowledge of how synapses alter their efficiency of communication is central to the understanding of learning and memory. The most extensively studied forms of synaptic plasticity are long-term potentiation (LTP) and its counterpart long-term depression (LTD) of AMPA receptor-mediated synaptic transmission. In the CA1 region of the hippocampus, it has been shown that LTP often involves a rapid increase in the unitary conductance of AMPA receptor channels. However, LTP can also occur in the absence of any alteration in AMPA receptor unitary conductance. In the present study we have used whole-cell dendritic recording, failures analysis and non-stationary fluctuation analysis to investigate the mechanism of depotentiation of LTP.

**Results:**

We find that when LTP involves an increase in unitary conductance, subsequent depotentiation invariably involves the return of unitary conductance to pre-LTP values. In contrast, when LTP does not involve a change in unitary conductance then depotentiation also occurs in the absence of any change in unitary conductance, indicating a reduction in the number of activated receptors as the most likely mechanism.

**Conclusions:**

These data show that unitary conductance can be bi-directionally modified by synaptic activity. Furthermore, there are at least two distinct mechanisms to restore synaptic strength from a potentiated state, which depend upon the mechanism of the previous potentiation.

## Background

Fast excitatory synaptic transmission in the central nervous system, which is mediated predominantly by the AMPA subtype of glutamate receptors, can undergo long-term bi-directional modifications in strength [1-3]. These persistent changes have been proposed to be key synaptic processes involved in learning and memory. The best characterised form of bi-directional synaptic plasticity is LTP / LTD of glutamatergic transmission in the CA1 region of the hippocampus. Although there has been intensive investigation of the induction and expression of LTP and LTD (see [4]), the precise molecular mechanisms by which these alterations in synaptic strength occur remain unclear. Possible mechanisms could be presynaptic such as changes in the release process (probability of neurotransmitter release or the amount of L-glutamate released from vesicles (e.g., [5-9]), and / or postsynaptic (e.g., [10-12]), such as a change in the number of AMPA receptors (AMPARs) available to bind transmitter, or in the properties of existing receptors (P_open_, activation or de-activation kinetics, desensitisation or single-channel conductance, γ).

Recent studies have provided information on the possible mechanisms underlying postsynaptic alterations in synaptic strength. There is evidence that AMPA receptors are inserted into the postsynaptic membrane during LTP [13-15] and removed from the synapse upon induction of LTD [16,17]. However, it has also been shown that LTP can involve a rapid increase in γ of existing AMPA receptors [18]. This could be caused by a Ca^2+^/calmodulin-kinase II (CaM-KII)-mediated phosphorylation of GluR1 which occurs during LTP [19], and which causes an increase in γ of GluR1 homomers in transfected cells [20]. Therefore, another potential mechanism for LTD could be a decrease in γ caused by a dephosphorylation of AMPARs.

We have recently used peak-scaled non-stationary fluctuation analysis (non-SFA; [21]) of synaptic currents recorded from CA1 pyramidal cell dendrites [18], to investigate the molecular basis of *de novo *LTD (LTD at naïve pathways; [22,23]). In these studies, LTD was never associated with a change in γ [17]. Indeed, evidence was presented that the underlying mechanism involved a reduction in the number of surface expressed AMPA receptors (LTD_N_).

In the present study we have investigated a second form of LTD known as depotentiation (DP), which is a reversal of pre-established LTP [24-27]. LTP can be associated with either an increase in γ (LTPγ) or no change in γ (LTP_N_) [18]. We were, therefore, interested to determine whether DP of LTPγ involved a decrease in γ and hence whether γ is a bi-directionally modifiable parameter. We find a reciprocal relationship between LTP and DP, such that DP of LTPγ invariably involves a restoration of the pre-LTP γ (DPγ) whereas DP of LTP_N _never involves a change in γ (DP_N_). These data show, firstly, that there are two distinct molecular mechanisms for the reduction of synaptic strength that are dependent on the nature of the preceding LTP and, secondly, that γ can indeed be bi-directionally modified in response to synaptic activity.

## Results

**Figure 1:**
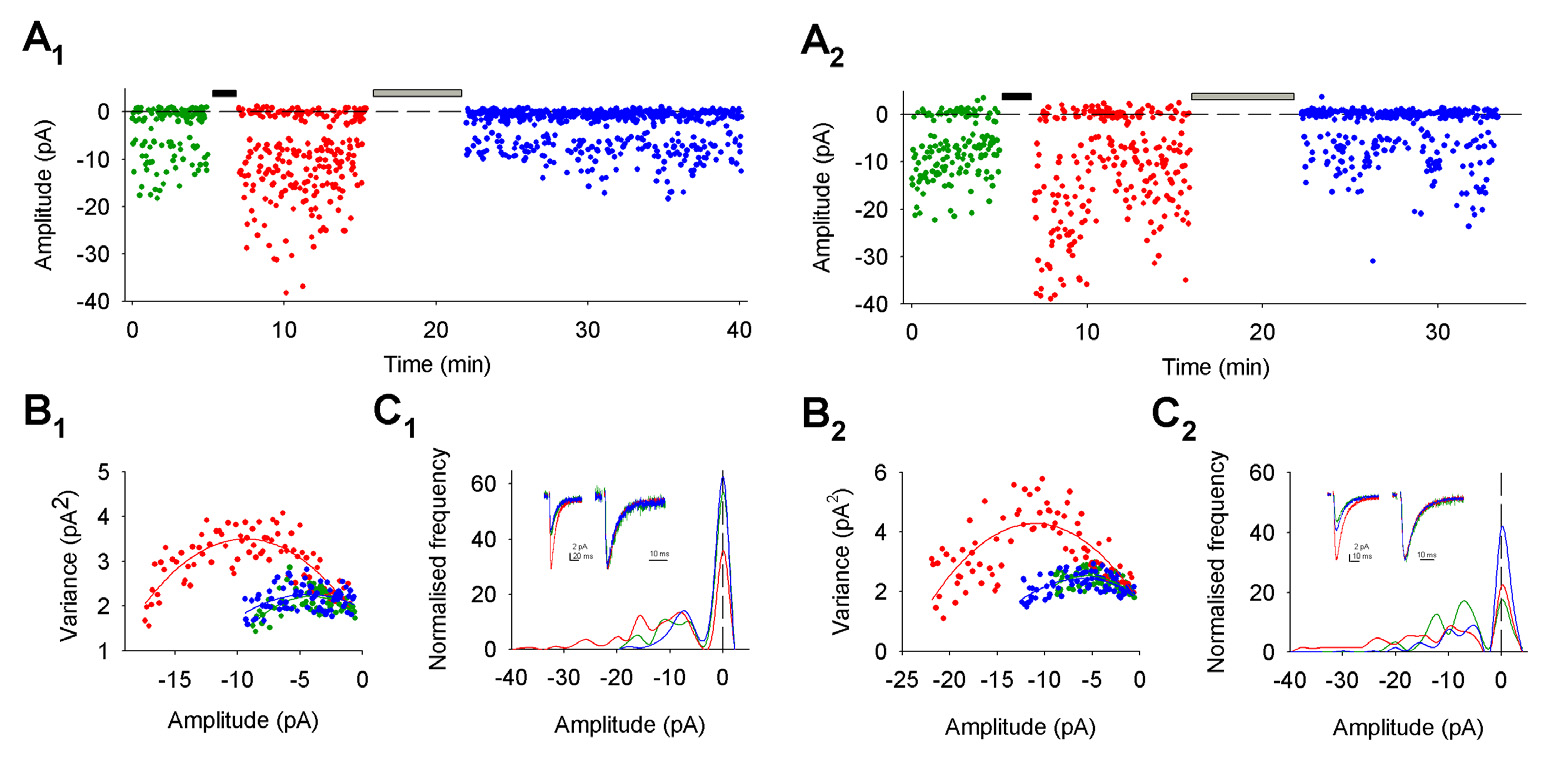
Bi-directional modification of AMPA receptor conductance (γ) during LTP (LTPγ) and DP (DP_γ_). (A1,2) Plot of amplitude *vs*. time from two representative experiments. Black bar represents LTP pairing, grey bar DP pairing protocol. Throughout the figure, green represents baseline, red LTP and blue DP. (B1,2) Current-variance relationship for baseline, LTP and DP (γ values for the same cells (pS): cell 1:baseline = 2.2, LTP = 6.5, DP = 3.7; cell 2: baseline = 4.5, LTP = 7.4, DP = 3.2). For this figure and in Figure 3, lines are parabolic fits of the data (see Methods). (C1,2) Inset. Mean EPSCs (average of all responses used for non-SFA) superimposed (left) and peak scaled (right). Amplitude histograms (bin width = 2 pA; number of trials: cell 1: N = 130 for baseline, N = 245 for LTP, N = 533 for DP; cell 2: N = 150 for baseline, N = 267 for LTP, N = 337 for DP; frequency normalised to the data set with the smallest number of observations for baseline, LTP and DP).

Using whole-cell recordings from the proximal apical dendrites of hippocampal CA1 pyramidal cells, minimal stimulation of nearby afferents evoked EPSCs that could be reliably resolved from failures (trials in which stimulation produced no synaptic response; Figure 1, see also Figure 3). These high resolution recordings enabled both a failures analysis to be performed [28] and, using non-SFA, an estimate of γ of synaptically-activated AMPARs to be obtained [18].

### LTP

To investigate the mechanism of DP, LTP was first induced in 18 cells by pairing afferent stimulation (baseline frequency) with a holding potential of 0 mV. This resulted in stable LTP (EPSC amplitude = 186 ± 16 % of baseline, n = 18).

In agreement with a previous study [18], cells fell into two groups with respect to changes (> 20%) in γ of AMPA receptor channels during LTP. In the majority of cases (11/18 cells), LTP was associated with an increase in γ (LTPγ; 246 ± 26% of baseline; range 136 – 363%). In the other 7 cells there was no increase in γ during LTP (100 ± 4 % of baseline; range 85 – 118 %), indicating that there was an increase in the functional number of channels activated (LTP_N_). As noted previously [18], there were no differences between the two groups of neurons with respect to a variety of baseline parameters.

### Depotentiation of LTPγ

**Figure 2:**
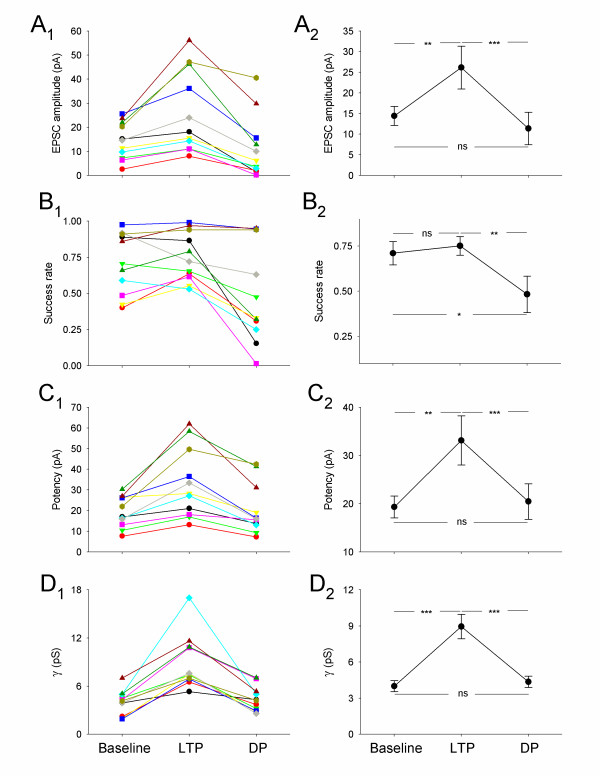
Summary data for all cells that exhibited DPγ. (A) Changes in mean EPSC amplitude for (A_1_) individual experiments, and (A_2_) pooled data. (B – D) Similar plots for (B_1 _& B_2_) success rate (1 – failure rate), (C_1 _& C_2_) potency and (D_1 _& D_2_) γ. In this, and Figure 4, ns = not significant, * P < 0.05, ** P < 0.01, *** P < 0.001).

Figure 1 shows two examples from the group of cells that exhibited LTPγ, as indicated by the change in the current-variance plot obtained from non-SFA (Figure 1B). As previously reported [18], the increase in γ was not associated with any change in EPSC kinetics (Figure 1C) indicating that AMPA receptor channel kinetics were not affected [29]. Failures analysis (Figure 1C) of this group of cells (Figure 2) revealed that LTP was associated with changes in success rate (1 – failure rate) in some cells (Figure 2B), and potency (mean EPSC amplitude excluding failures) in all cells (Figure 2C), as previously reported under these recording conditions [18,28].

For this group of cells, DP, induced by pairing stimulation (baseline frequency) with a holding potential of -40 mV, always resulted in a reversal of the γ increase, as indicated by the current-variance plot (Figure 1B; Figure 2D). Similar to the preceding LTPγ, this form of DP (DPγ) was also associated with no change in EPSC kinetics (τ_rise_: baseline = 1.6 ± 0.2 ms, LTPγ = 1.7 ± 0.3 ms, DPγ = 1.7 ± 0.2 ms, n = 11; τ_decay_: baseline = 8.3 ± 0.6 ms, LTPγ = 8.2 ± 0.6 ms, DPγ = 8.9 ± 0.7, n = 11; Figure 1C). Failures analysis showed that DPγ was associated with a decrease in success rate in most cells (Figure 2B) and a full reversal of the potency increase (Figure 2C). These data show that the primary mechanism for DP is the reversal of any increase in γ caused by LTP. Indeed, the changes in γ were sufficient to account for the potency changes during both LTPγ and DPγ. (In most cells the alterations in γ actually exceeded the potency changes. This is most likely due to an underestimate of the potency change due to dendritic filtering, which affects measurements at the peak of EPSCs greater than during the tail, from where the non-SFA estimates are obtained; see [18]).

### Depotentiation of LTP_N_

**Figure 3:**
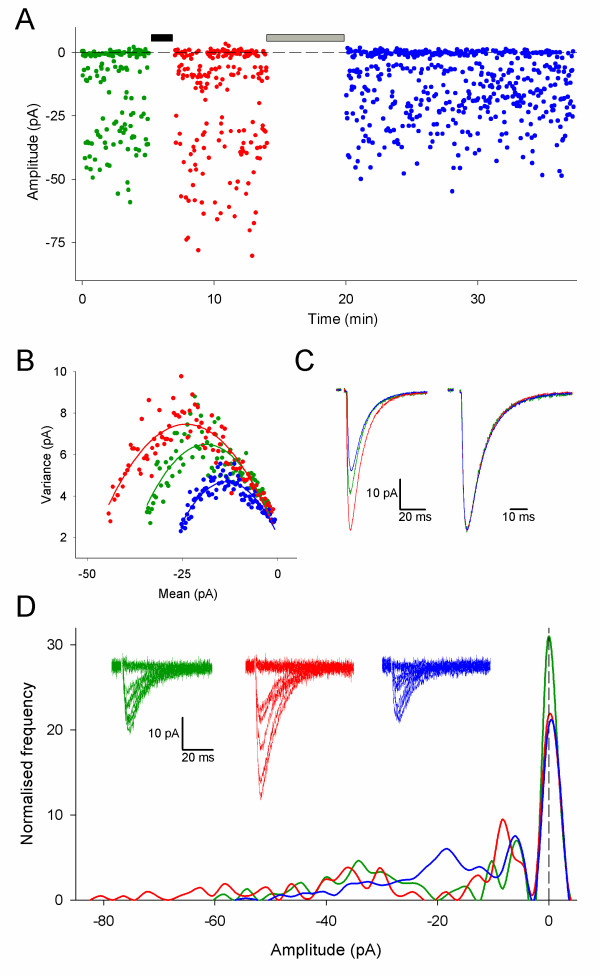
DP can occur without alterations in γ (DP_N_) but only when preceded by LTP that does not involve a change in γ (LTP_N_). (A) Plot of amplitude *vs*. time from a representative experiment. Black bar represents LTP pairing, grey bar DP pairing protocol. Throughout the figure, green represents baseline, red LTP and blue DP. (B) Current-variance relationship for baseline, LTP and DP (γ values for this cell (pS): baseline = 6.5, LTP = 6.7, DP = 6.0). (C) Mean EPSCs (average of all responses used for non-SFA) superimposed (left) and peak scaled (right). (D) Amplitude histogram (bin width = 2 pA; N = 151 for baseline, N = 211 for LTP, N = 513 for DP) for baseline, LTP and DP. Inset: 10 consecutive responses for baseline, LTP and DP.

**Figure 4:**
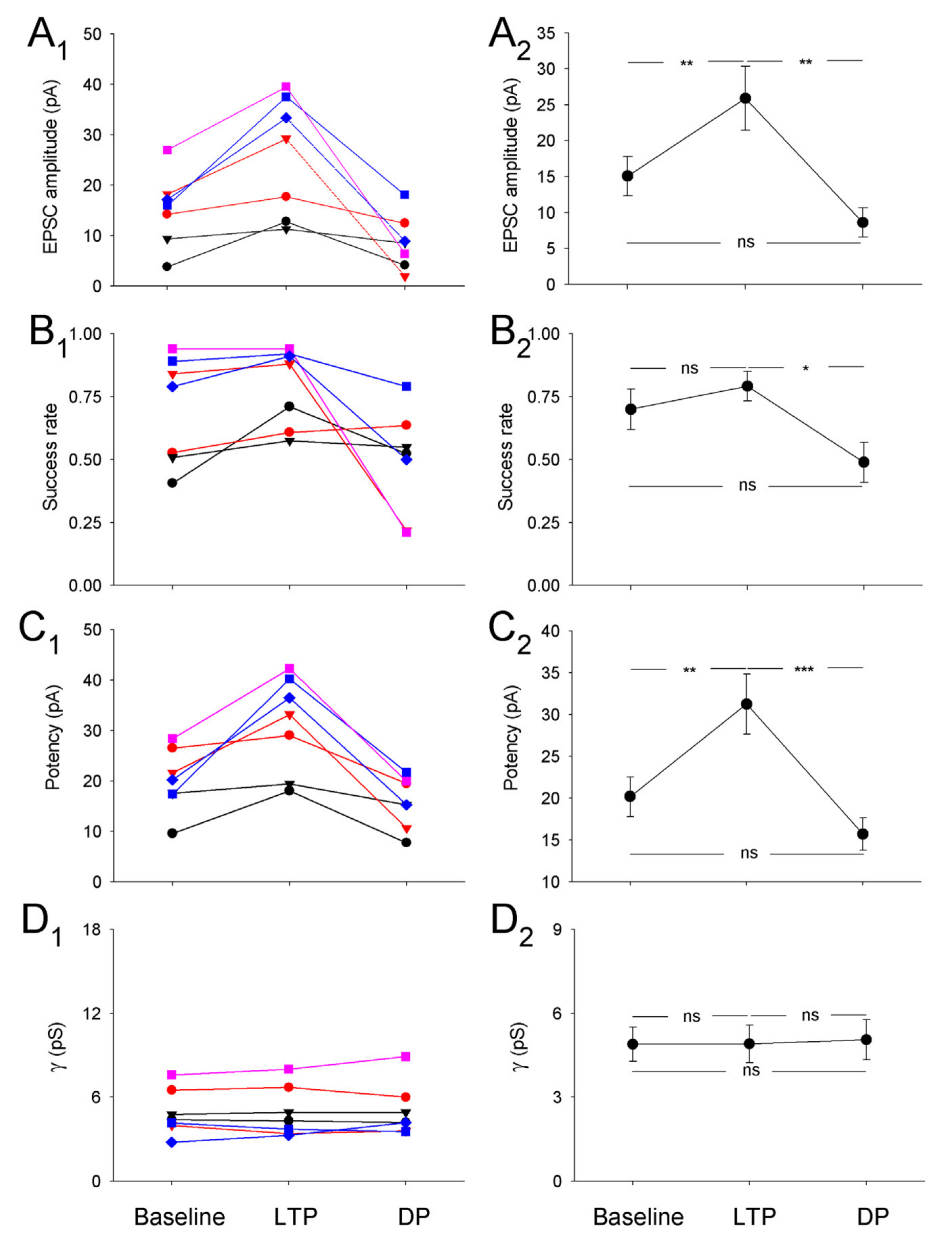
Summary data for all cells exhibiting DP_N_. (A) Changes in mean EPSC amplitude for (A_1_) individual experiments, and (A_2_) pooled data. (B – D) Similar plots for (B_1 _& B_2_) success rate, (C_1 _& C_2_) potency and (D_1 _& D_2_) γ.

Figure 3 shows an example from the group of cells that exhibited no change in γ during LTP (LTP_N_), as indicated by the current-variance plot (Figure 3B). The change in EPSC amplitude for LTP_N _neurons (Figure 4A) was similar to that for LTPγ neurons (Figure 2A). Failures analysis (Figure 3D) of this group of cells (Figure 4) revealed that LTP was associated with changes in success rate in some cells (Figure 4B), and potency in most cells (Figure 4C; P < 0.01), as previously reported under these recording conditions [18,28].

In contrast to DPγ, DP in these cells was never associated with a change in γ (DP_N_; Figure 3B, Figure 4D). There was also no change in EPSC kinetics with LTP or DP (τ_rise_: baseline = 1.9 ± 0.4 ms, LTP_N _= 2.0 ± 0.3 ms, DP_N _= 1.8 ± 0.3 ms, n = 7; τ_decay_: baseline = 9.7 ± 0.7 ms, LTP_N _= 9.7 ± 0.6 ms, DP_N _= 10.1 ± 0.9, n = 7; Figure 3C). Failures analysis of LTP_N _and DP_N _(Figure 3D, Figure 4) showed similar changes to the LTPγ group of cells in EPSC amplitude (Figure 4A), success rate (Figure 4B) and potency (Figure 4C). These data suggest that there is a second mechanism for DP, not involving a decrease in γ, which co-exists at CA1 synapses. Therefore, there are two mechanisms for the expression of DP that depend upon the expression mechanism of the previous potentiation.

### Role of NMDA receptors in depotentiation

**Figure 5:**
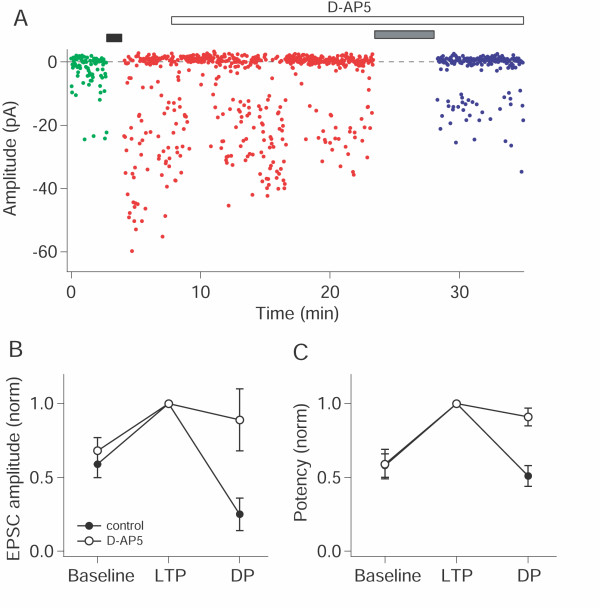
NMDA receptor-dependence of DP. (A) Plot of amplitude *vs*. time from an experiment in which the DP induction protocol was delivered in the presence of D-AP5 (50 μM). Black bar represents LTP pairing, grey bar DP pairing protocol. (B) Changes in mean EPSC amplitude for normalised pooled data. Filled symbols: control (n = 5); open symbols: D-AP5 (n = 4). (C) Changes in potency for normalised pooled data (symbols as in B). For the analysis of LTP, amplitude and potency were measured for the 200 trials immediately preceding the DP pairing protocol.

It has been shown previously that DP at CA1 synapses may be blocked either by NMDA receptor antagonists [26] or by mGlu receptor antagonists [27] and that this may depend on previous history [30]. In the present study we wished to focus on NMDA receptor-dependent synaptic plasticity and therefore used pairing protocols designed to activate NMDA receptors sufficiently to, firstly, induce NMDA receptor-dependent LTP and, secondly, to induce NMDA receptor-dependent DP. To verify that we were indeed investigating NMDA receptor-dependent DP we performed a series of experiments using the NMDA receptor antagonist D-AP5 interleaved with control experiments. Following the induction of LTP, D-AP5 (50 μM) was bath applied for 15 minutes before delivering the DP induction stimulus. Whilst DP was induced in the control experiments (25 ± 11% of baseline, n = 5; p < 0.05) it was blocked by D-AP5 (89 ± 21%, n = 4; Figure 5).

### Relationship of changes in success rate, potency and γ to the magnitude of DP

**Figure 6:**
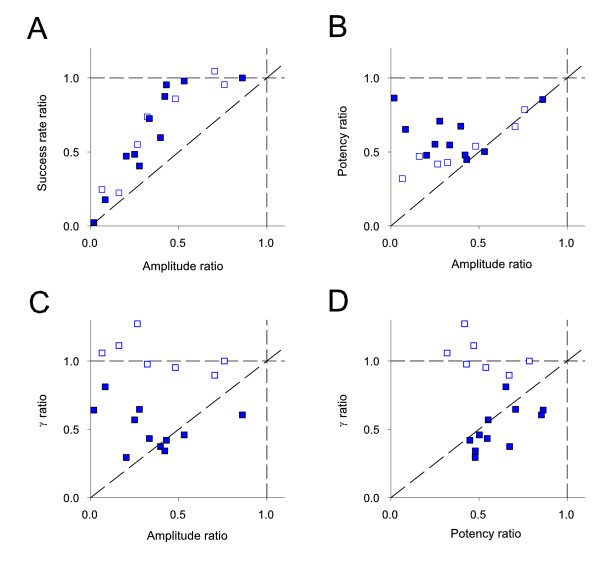
Further analysis of DP experiments. (A) Success rate ratio (DP / preceding LTP) *vs*. amplitude ratio for individual DP experiments (open symbols represent experiments for which γ did not change, closed symbols for experiments in which there was a change in γ). These symbol codes apply to the rest of the figure. (B) Potency ratio *vs*. amplitude ratio. (C) γ ratio *vs*. amplitude ratio. (D) γ ratio *vs*. potency ratio.

To gain further insights into the underlying mechanisms of DP we compared changes in EPSC amplitude, success rate, potency and γ for the individual experiments (Figure 6). A decrease in success rate indicates a reduction in probability of transmitter release (Pr) and/or a reduction in the number of functional synapses (n). A decrease in potency indicates a reduction in quantal amplitude (postsynaptic response to the release of a single quantum of transmitter, q) or, if multiple synapses are activated, a reduction in Pr or n.

In both types of neuron (i.e., LTPγ and LTP_N_), a small depression of less than 50 % (to 71 ± 7% of baseline; n = 4) was associated with no change in success rate (Figure 6A; success rate ratio = 1.00 ± 0.01) but an equivalent decrease in potency (Figure 6B; potency ratio = 0.70 ± 0.08), as is also observed for *de novo *LTD [17]). This indicates that the depression in these cells was associated primarily with a decrease in q. For larger depressions (to 26 ± 4 % of baseline; n = 14) there was also a marked decrease in success rate (success rate ratio = 0.52 ± 0.08; P < 0.01 *vs *success rate ratio for the group with DP < 50%) as well as a decrease in potency (potency ratio = 0.54 ± 0.04). This suggests that in these cells there was an additional decrease in Pr or n. Therefore, for DPγ, the change in γ did not fully account for the amplitude change in every cell (Figure 6C) but did account for the potency change (Figure 6D).

## Discussion

In this study we have shown that there are two mechanisms for NMDA receptor-dependent DP, a reduction in γ (DPγ) and a decrease in the number of activated AMPA receptors (DP_N_). As reported previously for different data sets from this age of rats [9,18] there are two forms of LTP; in approximately two-thirds of neurons LTP was associated with an increase in γ (LTPγ) whilst in the remainder there was no change in γ (LTP_N_). In the present study we saw a similar proportion of LTP expressed by changes in γ versus N. Strikingly, we observed a precise relationship between the mechanism of DP and the form of preceding LTP; LTPγ was always reversed by DPγ, and LTP_N _was always reversed by DP_N_.

In some experiments, induction of DP caused a decrease in EPSC amplitude below the initial baseline. This is most likely due to the simultaneous induction of DP and *de novo *LTD since in slices taken from juvenile animals, *de novo *LTD is readily induced by this [17] and other [22,23] induction protocols. This is in contrast to previous experiments using adult tissue in which DP induction depressed synaptic responses only as far as the initial baseline and where the same induction protocol was unable to induce *de novo *LTD [27]. Analysis of the mechanism of *de novo *LTD under the present experimental conditions demonstrated that it was associated with no change in γ [17]. Therefore the coexistence of DP and *de novo *LTD does not interfere with the analysis of DPγ.

In addition, there is sometimes a small, gradual run-down of synaptic responses observed in minimal stimulation experiments using two-week-old animals [17] see also [31]. Whilst this effect tends to exaggerate changes in amplitude and success rate during DP in some neurons, it does not significantly interfere with estimates of potency or γ (see [17]).

### Mechanisms underlying DP_N_

What might be the mechanism underlying DP that is not associated with a decrease in γ (i.e., DP_N_)? It is unlikely that this type of depression is due to a change in channel kinetics because there was no change in EPSC kinetics (see [18,29]). Therefore the mechanism is most likely a reduction in the number of activated AMPARs. This could be due to a presynaptic mechanism such as a reduction in release probability, the L-glutamate content of vesicles or the amount of L-glutamate discharged during fusion. Indeed there is evidence that some forms of LTD are expressed presynaptically [5,35]. Postsynaptic mechanisms for a reduction in the number of activated AMPA receptors include a reduction in their P_open _[36]or in the physical number of receptors present in the postsynaptic membrane [37].

**Figure 7:**
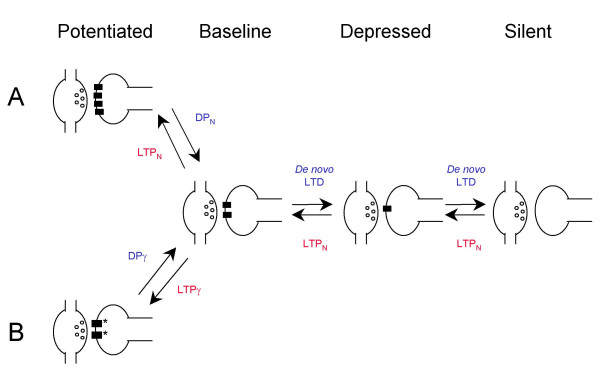
A model for the mechanisms of DP and *de novo *LTD. Rectangles represent AMPARs, open circles transmitter vesicles. (A) DP is due to a reduction in the number of surface-expressed AMPARs (DP_N_) when preceded by LTP_N_. Depression below the original baseline is due to a further reduction in AMPA receptor number causing a complete loss of postsynaptic receptors at some synapses. *De novo *LTD [17] is due to the same mechanism. (B) DP is due to a reduction in γ (DPγ) when preceded by LTPγ.

The present observations for DP_N _are indistinguishable from those that we and others have reported recently for *de novo *LTD [17,38]. For example, we showed that similar effects were obtained using the postsynaptic injection of a peptide (pep2m) that disrupts the interaction between NSF and GluR2 [39-42]. The effects of pep2m and those of *de novo *LTD were mutually occlusive, indicating a convergence of mechanisms. These data argue strongly for a postsynaptic mechanism of expression. Furthermore, since pep2m causes the removal of AMPA receptors from the membrane surface, as determined immunocytochemically [38,42], it is most likely that *de novo *LTD is due to the physical elimination of synaptic AMPA receptors. Other evidence for a postsynaptic mechanism for *de novo *LTD includes a reduction in the postsynaptic sensitivity to glutamate [43,44], the dephosphorylation of serine 845 of the GluR1 subunit [45,46] and a rapid internalisation of AMPA receptors [47] associated with LTD. Therefore, by analogy, we feel that the postsynaptic removal of AMPA receptors is also the most likely explanation for DP_N _(Figure 7A). Accordingly, a reduction in AMPA receptor number would account for the changes in potency without changes in success rate observed with modest DP_N_. The removal of an entire synaptic complement of AMPA receptors would explain the additional change in success rate seen with large depressions associated with DP_N _in some cells.

### Mechanisms underlying DP_γ_

A number of possible underlying mechanisms could account for the change in γ during LTPγ and DPγ. Non-SFA cannot distinguish between 1) a change from a single low conductance state to a single high conductance state, 2) changes in open times within a burst and, 3) changes in the proportion of time spent in different conductance states. Since AMPA receptors are known to have multiple conductance states [32,33] we have postulated that the proportion of time spent in different conductance states is the modifiable parameter [18]. Such a change is detectable with the type of analysis that we have used, which provides a weighted mean of the various sub-conductance states [48]. Independent support for this hypothesis is provided by a study which shows that phosphorylation of GluR1 at serine 831 increases the proportion of time AMPA receptors spend in high conductance states, as determined by single channel recording [20]. Indeed, phosphorylation of this residue occurs during LTP [19]. Thus a possible mechanism of DPγ is the dephosphorylation of serine 831 [45], perhaps involving calcineurin [49], resulting in a lower proportion of time AMPA receptors spend in the higher conductance states (Figure 7B).

Other theoretical possibilities exist to explain DPγ. For example, the silencing of synapses close to the patch electrode leaving more distant synapses contributing a lower net γ due to electrotonic filtering, or a decrease in trial-to-trial asynchrony of transmitter release. We believe that these possibilities are unlikely because in every cell in which *de novo *LTD was induced there was never a change in γ [17]. If such possibilities were likely, statistically one would expect similar changes to occur for both *de novo *LTD and DP. Moreover, we have also investigated these possibilities using our standard compartmental model [29]. This shows that 1) the electrotonic filtering required to achieve an artefactual decrease in γ would have a pronounced effect on τ_decay _which was never observed experimentally, and 2) pronounced changes in asynchrony necessary to cause an artefactual change in γ cause large deviations from the parabolic relationship in the current-variance plot (unpublished observations) and alterations in τ_rise _[29], also never observed experimentally. Another possibility is that alterations in vesicle fusion pore dynamics leading to substantial changes in the peak and time-course of cleft glutamate are caused by synaptic plasticity [7]. Such changes could differentially affect estimates of γ [50], however they would be associated with substantial changes in τ_rise _and τ_decay _[7], which were never observed experimentally.

## Conclusions

In summary, we have shown that there are two distinct molecular mechanisms for the reduction of synaptic strength. Although previous studies have provided evidence that LTD is associated with dephosphorylation of serine 845 on GluR1 [45] and internalisation of AMPA receptors [16,17] it is not known whether this represents two separate mechanisms or two components of the same process. For example, dephosphorylation of GluR1 could drive the internalisation of AMPA receptors. Here we show, for the first time, the co-existence of two distinct mechanisms for the expression of DP, using functional criteria under identical experimental conditions. The relationship between the two mechanisms is critically dependent upon the recent experience of the synapse, which may be governed by the phosphorylation state of the AMPA receptor complement [45]. Further work is now required to elucidate the relationship between these two fundamental mechanisms for modulating synaptic strength and the precise molecular mechanisms involved in each form of plasticity.

## Methods

### Electrophysiology

Hippocampal slices (400 μm) were obtained from 12–15 day old rats and perfused with an extracellular solution containing (in mM): 124 NaCl, 3 KCl, 1.25 NaHPO_4_, 26 NaHCO_3_, 2 CaCl_2_, 1 MgSO_4_, 15 glucose, 2 ascorbic acid, 0.05 picrotoxin, saturated with 95% O_2 _/ 5% CO_2_, at room temperature (23–25°C). Individual dendrites were visualised using infrared illumination and DIC optics and approached under visual control. Whole-cell dendritic recordings of synaptic currents were obtained at a holding potential of -70 mV using patch electrodes (6–10 MΩ) filled with a solution containing (in mM): 135 CsMeSO_4_, 8 NaCl, 10 HEPES, 0.5 EGTA, 4 Mg-ATP, 0.3 Na-GTP, 5 QX-314, pH 7.25, 285 mOsm. Schaffer collateral-commissural fibers were stimulated at 0.5 Hz using a platinum monopolar or concentric bipolar electrode, which was positioned 20–40 μm from the dendrite parallel to the input pathway. The stimulus intensity was set to evoke some failures to enable a failures analysis to be performed and to ensure that the majority of EPSCs were, for any given trial, evoked by release from a single site. However, to elicit sufficient EPSCs to obtain a baseline estimate of γ before "washout" of LTP it was usually necessary to set the success rate fairly high (usually above 50%). As a result it is likely that multiple release sites contributed to the recordings. LTP was induced by pairing 40–60 stimuli (baseline frequency) with a holding potential of 0 mV. DP was induced following the induction of stable LTP by 100–200 stimuli (baseline frequency) at -40 mV holding potential. All recordings were made using an Axopatch 1B amplifier, signals were filtered at 5 kHz (8 pole Bessel filter), digitised at 10 kHz and stored on computer. EPSC amplitude and input resistance were analysed and displayed on-line using the 'LTP' program [51]). Series resistance was estimated by measuring the peak amplitude of the fast whole-cell capacitance current in response to a -1 mV step applied to the cell during each sweep. The amplitude was estimated by fitting the capacitance transient with a double exponential (from 0.5 ms after the peak) and determining the current at the beginning of the step. Series resistance was stable throughout recordings (series resistance values [MΩ]: baseline = 42 ± 3, LTP = 40 ± 3, DP = 43 ± 3, n = 17).

### Analysis

Non-SFA was performed as described previously [18]. Briefly, synaptic currents were aligned by their point of maximal rise, and averaged. The average response waveform was scaled to the peak, subtracted from individual responses and the variance of the decays calculated. The variance was plotted *vs*. the mean current amplitude and the single channel current was estimated by fitting the data to: σ^2 ^= iI - I^2^/N + b_l_, where σ^2 ^is the variance, I is the mean current, N is the number of channels activated at the peak, i is the single channel current and b_l _is the background variance. The single channel conductance (γ) is then γ = i/V, where V is the driving force (holding potential – assumed reversal potential of 0 mV). Response amplitude was measured in two ways. For failures, amplitude was estimated by measuring the difference between the average current over two time windows of equal length, one immediately before the stimulus artefact and the other centred on the peak of the mean EPSC. For estimation of the amplitude of successes the peak amplitude over a set time window was determined. Failures were identified visually, and potency was calculated as the mean EPSC amplitude excluding failures. For analysis of EPSC kinetics, rise time was estimated by the time-constant of a single exponential fit of the rising phase of the mean EPSC waveform. For decay, the time-constant of the single exponential fit to the decay phase was used. For display of individual EPSC traces in the figures, the stimulus artefact was digitally subtracted using an average of identified failures. All histograms are represented as smoothed line plots (SigmaPlot ver 5.0). Data are expressed as % of baseline (i.e., 100 % = no change). Statistical significance was assessed using the Student's *t*-test (one or two-tailed, paired or unpaired as appropriate; *P *< 0.05 as significant). All values are expressed as mean ± s.e.m.

## Authors' contributions

AL performed most of the electrophysiology. MJP and MAW also contributed some recordings. PM provided some information that guided the work. TB performed the modelling that provided the theoretical frame-work. JI helped with the design and analysis of experiments. GLC coordinated the work and contributed to its design. All authors read and approved the manuscript.

## References

[B1] Bliss TV, Collingridge GL (1993). A synaptic model of memory: long-term potentiation in the hippocampus. Nature.

[B2] Malenka RC, Nicoll RA (1999). Long-term potentiation--a decade of progress?. Science.

[B3] Bear MF, Abraham WC (1996). Long-term depression in hippocampus. Annu Rev Neurosci.

[B4] Bliss TV, Collingridge GL, Morris RG (2003). Long-term potentiation: enhancing neuroscience for 30 years. Phil Soc Trans.

[B5] Stevens CF, Wang Y (1994). Changes in reliability of synaptic function as a mechanism for plasticity. Nature.

[B6] Malgaroli A, Ting AE, Wendland B, Bergamaschi A, Villa A, Tsien RW, Scheller RH (1995). Presynaptic component of long-term potentiation visualized at individual hippocampal synapses. Science.

[B7] Choi S, Klingauf J, Tsien RW (2000). Postfusional regulation of cleft glutamate concentration during LTP at 'silent synapses'. Nat Neurosci.

[B8] Emptage NJ, Reid CA, Fine A, Bliss TV (2003). Optical quantal analysis reveals a presynaptic component of LTP at hippocampal Schaffer-associational synapses. Neuron.

[B9] Palmer MJ, Isaac JT, Collingridge GL (2004). Multiple, developmentally regulated expression mechanisms of long-term potentiation at CA1 synapses. J Neurosci.

[B10] Davies SN, Lester RA, Reymann KG, Collingridge GL (1989). Temporally distinct pre- and post-synaptic mechanisms maintain long-term potentiation. Nature.

[B11] Nicoll RA, Malenka RC (1995). Contrasting properties of two forms of long-term potentiation in the hippocampus. Nature.

[B12] Mainen ZF, Jia Z, Roder J, Malinow R (1998). Use-dependent AMPA receptor block in mice lacking GluR2 suggests postsynaptic site for LTP expression. Nat Neurosci.

[B13] Hayashi Y, Shi SH, Esteban JA, Piccini A, Poncer JC, Malinow R (2000). Driving AMPA receptors into synapses by LTP and CaMKII: requirement for GluR1 and PDZ domain interaction. Science.

[B14] Lu W, Man H, Ju W, Trimble WS, MacDonald JF, Wang YT (2001). Activation of synaptic NMDA receptors induces membrane insertion of new AMPA receptors and LTP in cultured hippocampal neurons. Neuron.

[B15] Pickard L, Noel J, Duckworth JK, Fitzjohn SM, Henley JM, Collingridge GL, Molnar E (2001). Transient synaptic activation of NMDA receptors leads to the insertion of native AMPA receptors at hippocampal neuronal plasma membranes. Neuropharmacology.

[B16] Beattie EC, Carroll RC, Yu X, Morishita W, Yasuda H, von Zastrow M, Malenka RC (2000). Regulation of AMPA receptor endocytosis by a signaling mechanism shared with LTD. Nat Neurosci.

[B17] Luthi A, Chittajallu R, Duprat F, Palmer MJ, Benke TA, Kidd FL, Henley JM, Isaac JT, Collingridge GL (1999). Hippocampal LTD expression involves a pool of AMPARs regulated by the NSF-GluR2 interaction. Neuron.

[B18] Benke TA, Luthi A, Isaac JT, Collingridge GL (1998). Modulation of AMPA receptor unitary conductance by synaptic activity. Nature.

[B19] Barria A, Muller D, Derkach V, Griffith LC, Soderling TR (1997). Regulatory phosphorylation of AMPA-type glutamate receptors by CaM-KII during long-term potentiation. Science.

[B20] Derkach V, Barria A, Soderling TR (1999). Ca2+/calmodulin-kinase II enhances channel conductance of alpha-amino-3-hydroxy-5-methyl-4-isoxazolepropionate type glutamate receptors. Proc Natl Acad Sci U S A.

[B21] Traynelis SF, Silver RA, Cull-Candy SG (1993). Estimated conductance of glutamate receptor channels activated during EPSCs at the cerebellar mossy fiber-granule cell synapse. Neuron.

[B22] Dudek SM, Bear MF (1992). Homosynaptic long-term depression in area CA1 of hippocampus and effects of N-methyl-D-aspartate receptor blockade. Proc Natl Acad Sci U S A.

[B23] Mulkey RM, Malenka RC (1992). Mechanisms underlying induction of homosynaptic long-term depression in area CA1 of the hippocampus. Neuron.

[B24] Barrionuevo G, Schottler F, Lynch G (1980). The effects of repetitive low frequency stimulation on control and "potentiated" synaptic responses in the hippocampus. Life Sci.

[B25] Staubli U, Lynch G (1990). Stable depression of potentiated synaptic responses in the hippocampus with 1-5 Hz stimulation. Brain Res.

[B26] Fujii S, Saito K, Miyakawa H, Ito K, Kato H (1991). Reversal of long-term potentiation (depotentiation) induced by tetanus stimulation of the input to CA1 neurons of guinea pig hippocampal slices. Brain Res.

[B27] Bortolotto ZA, Bashir ZI, Davies CH, Collingridge GL (1994). A molecular switch activated by metabotropic glutamate receptors regulates induction of long-term potentiation. Nature.

[B28] Isaac JT, Luthi A, Palmer MJ, Anderson WW, Benke TA, Collingridge GL (1998). An investigation of the expression mechanism of LTP of AMPA receptor-mediated synaptic transmission at hippocampal CA1 synapses using failures analysis and dendritic recordings. Neuropharmacology.

[B29] Benke TA, Luthi A, Palmer MJ, Wikstrom MA, Anderson WW, Isaac JT, Collingridge GL (2001). Mathematical modelling of non-stationary fluctuation analysis for studying channel properties of synaptic AMPA receptors. J Physiol.

[B30] Montgomery JM, Madison DV (2002). State-dependent heterogeneity in synaptic depression between pyramidal cell pairs. Neuron.

[B31] Xiao MY, Wasling P, Hanse E, Gustafsson B (2004). Creation of AMPA-silent synapses in the neonatal hippocampus. Nat Neurosci.

[B32] Jahr CE, Stevens CF (1987). Glutamate activates multiple single channel conductances in hippocampal neurons. Nature.

[B33] Cull-Candy SG, Usowicz MM (1987). Multiple-conductance channels activated by excitatory amino acids in cerebellar neurons. Nature.

[B34] Bolshakov VY, Siegelbaum SA (1994). Postsynaptic induction and presynaptic expression of hippocampal long-term depression. Science.

[B35] Oliet SH, Malenka RC, Nicoll RA (1997). Two distinct forms of long-term depression coexist in CA1 hippocampal pyramidal cells. Neuron.

[B36] Banke TG, Bowie D, Lee H, Huganir RL, Schousboe A, Traynelis SF (2000). Control of GluR1 AMPA receptor function by cAMP-dependent protein kinase. J Neurosci.

[B37] Malinow R, Malenka RC (2002). AMPA receptor trafficking and synaptic plasticity. Annu Rev Neurosci.

[B38] Luscher C, Xia H, Beattie EC, Carroll RC, von Zastrow M, Malenka RC, Nicoll RA (1999). Role of AMPA receptor cycling in synaptic transmission and plasticity. Neuron.

[B39] Nishimune A, Isaac JT, Molnar E, Noel J, Nash SR, Tagaya M, Collingridge GL, Nakanishi S, Henley JM (1998). NSF binding to GluR2 regulates synaptic transmission. Neuron.

[B40] Osten P, Srivastava S, Inman GJ, Vilim FS, Khatri L, Lee LM, States BA, Einheber S, Milner TA, Hanson PI, Ziff EB (1998). The AMPA receptor GluR2 C terminus can mediate a reversible, ATP-dependent interaction with NSF and alpha- and beta-SNAPs. Neuron.

[B41] Song I, Kamboj S, Xia J, Dong H, Liao D, Huganir RL (1998). Interaction of the N-ethylmaleimide-sensitive factor with AMPA receptors. Neuron.

[B42] Noel J, Ralph GS, Pickard L, Williams J, Molnar E, Uney JB, Collingridge GL, Henley JM (1999). Surface expression of AMPA receptors in hippocampal neurons is regulated by an NSF-dependent mechanism. Neuron.

[B43] Kandler K, Katz LC, Kauer JA (1998). Focal photolysis of caged glutamate produces long-term depression of hippocampal glutamate receptors. Nat Neurosci.

[B44] Rammes G, Palmer M, Eder M, Dodt HU, Zieglgansberger W, Collingridge GL (2003). Activation of mGlu receptors induces LTD without affecting postsynaptic sensitivity of CA1 neurons in rat hippocampal slices. J Physiol.

[B45] Kameyama K, Lee HK, Bear MF, Huganir RL (1998). Involvement of a postsynaptic protein kinase A substrate in the expression of homosynaptic long-term depression. Neuron.

[B46] Lee HK, Kameyama K, Huganir RL, Bear MF (1998). NMDA induces long-term synaptic depression and dephosphorylation of the GluR1 subunit of AMPA receptors in hippocampus. Neuron.

[B47] Carroll RC, Beattie EC, von Zastrow M, Malenka RC (2001). Role of AMPA receptor endocytosis in synaptic plasticity. Nat Rev Neurosci.

[B48] Cull-Candy SG, Howe JR, Ogden DC (1988). Noise and single channels activated by excitatory amino acids in rat cerebellar granule neurones. J Physiol.

[B49] Zhuo M, Zhang W, Son H, Mansuy I, Sobel RA, Seidman J, Kandel ER (1999). A selective role of calcineurin aalpha in synaptic depotentiation in hippocampus. Proc Natl Acad Sci U S A.

[B50] Smith TC, Howe JR (2000). Concentration-dependent substate behavior of native AMPA receptors. Nat Neurosci.

[B51] Anderson WW, Collingridge GL (2001). The LTP Program: a data acquisition program for on-line analysis of long-term potentiation and other synaptic events. J Neurosci Methods.

